# Extranuclear Inheritance of Mitochondrial Genome and Epigenetic Reprogrammability of Chromosomal Telomeres in Somatic Cell Cloning of Mammals

**DOI:** 10.3390/ijms22063099

**Published:** 2021-03-18

**Authors:** Marcin Samiec, Maria Skrzyszowska

**Affiliations:** Department of Reproductive Biotechnology and Cryoconservation, National Research Institute of Animal Production, 32-083 Kraków, Poland; maria.skrzyszowska@iz.edu.pl

**Keywords:** cloned mammalian embryo, SCNT-derived progeny, mtDNA, nuclear–mitochondrial interaction, epigenetic reprogrammability, telomere shortening/attrition

## Abstract

The effectiveness of somatic cell nuclear transfer (SCNT) in mammals seems to be still characterized by the disappointingly low rates of cloned embryos, fetuses, and progeny generated. These rates are measured in relation to the numbers of nuclear-transferred oocytes and can vary depending on the technique applied to the reconstruction of enucleated oocytes. The SCNT efficiency is also largely affected by the capability of donor nuclei to be epigenetically reprogrammed in a cytoplasm of reconstructed oocytes. The epigenetic reprogrammability of donor nuclei in SCNT-derived embryos appears to be biased, to a great extent, by the extranuclear (cytoplasmic) inheritance of mitochondrial DNA (mtDNA) fractions originating from donor cells. A high frequency of mtDNA heteroplasmy occurrence can lead to disturbances in the intergenomic crosstalk between mitochondrial and nuclear compartments during the early embryogenesis of SCNT-derived embryos. These disturbances can give rise to incorrect and incomplete epigenetic reprogramming of donor nuclei in mammalian cloned embryos. The dwindling reprogrammability of donor nuclei in the blastomeres of SCNT-derived embryos can also be impacted by impaired epigenetic rearrangements within terminal ends of donor cell-descended chromosomes (i.e., telomeres). Therefore, dysfunctions in epigenetic reprogramming of donor nuclei can contribute to the enhanced attrition of telomeres. This accelerates the processes of epigenomic aging and replicative senescence in the cells forming various tissues and organs of cloned fetuses and progeny. For all the above-mentioned reasons, the current paper aims to overview the state of the art in not only molecular mechanisms underlying intergenomic communication between nuclear and mtDNA molecules in cloned embryos but also intrinsic determinants affecting unfaithful epigenetic reprogrammability of telomeres. The latter is related to their abrasion within somatic cell-inherited chromosomes.

## 1. Biotechnological Possibilities of Applying the Techniques of Somatic Cell Nuclear Transfer (SCNT) to Produce Cloned Mammalian Species

The somatic cell cloning technique is a method of embryonic genome engineering. Unlike animal transgenesis, it involves micromanipulation not of individual nuclear DNA genes but of the whole nuclear and/or mitochondrial genome of both interphase nuclear donor somatic cells and female germ cells (in vitro- or in vivo-matured oocytes arrested at metaphase II), which are used as recipients of exogenous genetic material. Of all mammalian cloning techniques, somatic cell cloning can result in producing the largest numbers of genetically identical individuals that are designated as clones. In the somatic cell cloning of mammals, nuclear donor cells are available in practically unlimited quantities. Tissue samples obtained by biopsy from adult animals or fetuses are composed of hundreds of thousands cells, which can be further multiplied/expanded in vitro. Furthermore, when cloning certain adult animals, tissue may be biopsied repeatedly to produce identical clones every time [1,2,3,4,5,6,7].

Animal cloning by somatic cell nuclear transfer (SCNT), which avoids the sexual reproduction pathway, offers the opportunity to obtain monogenetic offspring derived not only from adult animals of high genetic merit but also from genetically transformed (transgenic) specimens. Over the last 24 years, intra- and interspecies cloning via SCNT resulted in a fairly large number of transgenic and non-transgenic offspring, not only in various species or infertile interspecific hybrids (bastards) of domesticated animals, such as

(1) cattle [8,9,10,11,12,13,14];
(2) goats [6,15,16,17,18,19,20];
(3) sheep [21,22,23,24,25,26];
(4) pigs [27,28,29,30,31,32,33,34,35,36,37,38,39,40,41];
(5) equids—domestic horses [42,43,44,45,46] and mules [47];
(6) water buffaloes—Chinese swamp buffaloes [48,49] and Indian river/riverine buffaloes [50,51,52,53];
(7) one-humped or dromedary camels [7,54,55,56];
(8) two-humped or Bactrian camels [57];
(9) domestic cats [58,59,60,61,62,63];
(10) domestic dogs [64,65,66,67,68,69,70,71];
(11) polecat-ferrets [72];
(12) rabbits [73,74,75,76,77,78];
(13) mice [79,80,81,82,83,84];
(14) rats [85]; but also in several species of endangered or non-endangered wild mammals, such as
(15) gaur [86,87];
(16) mouflon [88];
(17) European red deer [89];
(18) African wild cat [90];
(19) Arabian sand cat [91];
(20) Eurasian gray wolf [92,93];
(21) coyote or prairie wolf [94];
(22) cynomolgus monkey, also known as Java macaque, crab-eating macaque, or long-tailed macaque—a catarrhine monkey from the family *Cercopithecidae* [95]; and even in the extinct subspecies of the Spanish/Iberian ibex:
(23) Pyrenean ibex, a wild goat known as bucardo [96].

Explanation of the mechanisms underlying intergenomic communication between nuclear and mitochondrial DNA molecules in cloned embryos and recognition/identification of the determinants affecting aberrant epigenetic reprogrammability of chromosomal telomeres will be suitable and reliable for resolving or reducing the imperfections in the generation of cloned embryos, conceptuses, and offspring by using SCNT technology. Moreover, the development of efficient strategies applied to the cryopreservation of nuclear donor somatic cells, nuclear-transferred oocytes reconstructed with somatic cells, and somatic cell-cloned embryos seems to be an inevitable progressive step contributing to the expedition of future large-scale attempts aimed to more successfully produce and multiply mammalian cloned offspring. The latter seems to be a sine qua non condition that allows one to more efficiently use SCNT-based assisted reproductive technology not only for transgenic, biotechnological, biomedical, and biopharmaceutical research but also for the ex situ conservation of biodiversity in both anthropogenic (agricultural) and non-anthropogenic (wild) ecosystems.

## 2. Dependence of Epigenetic Mechanisms Underlying Somatic Cell Nuclear Reprogramming and Intergenomic Communication between Nuclear and Mitochondrial DNA Fractions in Cloned Embryos on Various Approaches to Reconstruction of Enucleated Oocytes

In the reconstruction of enucleated oocytes (cytoplasts/ooplasts) by SCNT, the original genetic material is replaced with the somatic cell-inherited nuclear genome. Different approaches to SCNT are used to generate nuclear-transferred oocytes, i.e., oocytes reconstructed with somatic cell nuclei and the resultant cloned embryos (Table 1). The most common procedure is a relatively low-invasive method of SCNT based on the fusion of cytoplast–nuclear donor cell couplets that is induced by electric pulses [34,97,98,99,100,101] (Table 1). An alternative reconstruction method is a much more invasive microsurgical procedure, in which whole nuclear donor cells [102,103] or somatic-cell-derived karyoplasts [29,44,82,104,105] are microinjected directly into the cytoplasm of enucleated oocytes (Table 1). The karyoplast is a live membrane-bound structure formed as a result of mechanically induced lysis of the whole somatic cell. It contains the interphase cell nucleus or metaphase chromosomes that are surrounded only by a thin layer of the perinuclear cytoplasm (the so-called perikaryon) [27,105,106,107,108].

Whatever the method used, the reconstruction of ooplasts results in the combination and mingling (hybridization) of cytoplasmic environments of the ooplast and intact somatic cell or karyoplast isolated from the whole nuclear donor cell. As a result, a nuclear–cytoplasmic/nuclear–ooplasmic hybrid (i.e., cloned cybrid) is formed. This hybrid cell, formed by the hybridization of cytoplasmic microenvironments of the cells derived from two different developmental lines: gametogenic (germinal) and somatogenic (somatic), is referred to as a reconstructed or reconstituted oocyte or cybrid cloned zygote. As the mitotic cycle of nuclear donor somatic cells (artificially arrested at the G0 phase) is characterized by “latent” transcriptional activity, inhibited proliferative growth, and a slower metabolism of all organelles, the meiotic cycle of nuclear recipient oocytes also undergoes transient and reversible arresting at the metaphase II (MII) stage. At this stage of meiosis, the processes of advanced transcriptional suppression of genomic DNA take place as a result of attaining nuclear and ooplasmic maturity states. Proper coordination of the cytophysiological state of somatic cells or the karyoplasts isolated from them, and of the cytophysiological state of ooplasts during the reconstruction of cloned cybrids, results from the hybridization of the cytoplasmic environments of nuclear donor cells at the G0 phase of mitosis and of enucleated nuclear recipient oocytes at the MII stage of meiosis [27,98,101,109,110].

Techniques of enucleated oocyte reconstruction may largely affect molecular mechanisms of nuclear chromatin rearrangement, which include both its structural remodeling and epigenetic reprogramming of genomic DNA [99,102,104,107,111,112,113]. Hybridizing the cytoplasmic environment of two cells at different stages of the division cycle interferes with the cell cycle controlling mechanisms and carries the risk of abnormalities further into the development of the cybrid cloned zygote. However, not only does the proper selection of the cytophysiological states of somatic cells/karyoplasts and ooplasts during the reconstruction of cloned cybrids reduce genomic instability, rendering the genome less vulnerable to mutations, but it also reduces the degree of asynchrony in nuclear–cytoplasmic interactions and decreases the frequency of abnormal epigenome-dependent rearrangements of exogenous nuclear chromatin [114,115,116,117,118,119,120].

In contrast to electrofusion, intraooplasmic microinjection of karyoplasts allows for the selective removal of a large part of the cytoplasm of nuclear donor cells, thus enabling relative thinning of the remnants of the somatic cell cytoplasm in a cytosolic microenvironment of the ooplast and early zygote. The direct consequence of this is that the adverse effect of cytoplasmic components of the somatic cell on remodeling and reprogramming of the transferred somatic cell nucleus, and thereby on the development of the reconstituted embryo, is avoided. Where nuclei of relatively small-diameter somatic cells are transplanted (e.g., cumulus oophorus cells, mural granulosa cells, and serum-starved fibroblast cells), the method of choice is the intraooplasmic microinjection of karyoplasts or whole nuclear donor cells [80,98,102,103,104,112,121,122]. Taking into account the above-mentioned finding, the in vitro developmental potential of cloned pig embryos that had been reconstructed by direct intraooplasmic microinjection of somatic cell-descended karyoplasts or whole tiny somatic cells was shown to be relatively higher in relation to cloned embryos produced by the electrofusion of somatic cell–ooplast couplets [29,102,107]. The small diameter of the above types of somatic cells is the reason for a considerably reduced contact surface area with the plasmalemma of enucleated oocytes (oolemma), which reduces the percentage of fused ooplast–nuclear donor cell complexes. In turn, the direct microinjection of karyoplasts or whole small-diameter somatic cells into the cytoplasm of enucleated oocytes avoids technical problems (resulting from inadequate adhesion of plasma membranes), which have the greatest limiting effect on the efficiency of electrofusion of nuclear donor cells with cytoplasts [102,103,104,122].

The direct microinjection of somatic cell nuclei into the cytoplasm of enucleated oocytes has the added advantage of being the “cleanest” of all nuclear transplantation methods. It requires no physicochemical transducers, which often have adverse effects by reducing the in vitro developmental potential of mammalian cloned embryos. For the cell electrofusion technique, all components of the donor cell (both nuclear and cytoplasmic components: organelles and cytoskeletal elements) become an integral part of the oocyte. In contrast, for intraooplasmic microinjection of karyoplasts, plasmalemma and the vast majority of the cytoplasmic material of the nuclear donor cell is rejected following cell lysis. Therefore, only trace amounts of residual cytoplasm, in the form of a narrow rim of membrane-bound protoplasm around the cell nucleus, are introduced as a small karyoplast into the enucleated oocyte. This is of prime importance in some studies that examine nuclear–cytoplasmic interactions in mammalian cloned cybrids [29,44,81,82,104,107,111,122].

The basic paradigm underlying the somatic cell cloning of mammals is the scientific thesis that the donor cell nucleus has to be completely reprogrammed epigenetically by specific factors of the oocyte’s origin in order to support the development of the cybrid cloned zygote to term. A considerable portion of the protein nucleoplasmic (karyolymphatic) factors and cytosolic factors of the somatic cell, which are engaged directly or indirectly in the mechanisms underlying epigenetic reprogramming of donor cell genome, is associated with nuclear chromatin. The qualitative and quantitative composition of these factors within the somatic cell changes together with progressing cytodifferentiation. When the whole donor cell is fused with the enucleated oocyte, those specific factors of somatic cell are also transferred into the cytoplasm of the nuclear recipient oocyte. As a result of this, they may block the endogenous oocyte factors from supporting proper remodeling and reprogramming the epigenetic profile, which is characteristic of a foreign nucleus of a terminally differentiated somatic cell, toward an epigenetic status typical of the nucleus of totipotent stem cells such as the zygote [28,102,123,124,125,126,127]. Exogenous nucleoplasmic and cytoplasmic factors derived from the nuclear donor cell, which are responsible for modulating the epigenetic status of genomic DNA, are incorporated together with oocyte mRNA transcripts and proteins, into the remodeled nucleus of the somatic cell (the so-called pseudo-pronucleus). The pseudo-pronucleus is formed following artificial activation of the embryonic developmental program of the reconstructed oocyte [1,128,129,130,131,132,133,134,135,136,137]. In turn, an overabundance of the somatic cell-derived agents modulating the epigenetic profile of the donor nucleus may remarkably reduce the concentration and activity of the oocyte’s epigenetic factors. Thus, it may diminish the incidence of complete epigenetic reprogramming of transcriptional activity of the somatic cell nucleus in the developing cloned embryo [112,132,133,134,135,136,137,138,139,140,141].

## 3. Inheritance of the Mitochondrial Genome and Intergenomic Communication between Mitochondrial and Nuclear DNA Fractions during the Development of Cloned Embryos

The increased competence of the oocyte cytoplasm for epigenetic remodeling and reprogramming the somatic cell-inherited nuclear and mitochondrial genomes in cybrid cloned zygotes is a sine qua non condition for correctly inducing the developmental program specific for mammalian SCNT embryos [142,143,144,145,146,147,148,149,150,151,152,153,154].

Mitochondria are semiautonomous organelles that contain their own genetic material in the form of double-stranded (α-helix) circular DNA molecules (mtDNAs) of about 16,300–16,500 base pairs (bp). The mitochondrial DNA encodes 13 proteins, 22 tRNAs, and 2 rRNAs. Up to 95% of proteins, which are the products of the cytoplasmic translation system encoded by nuclear DNA, are involved in biogenesis and cytophysiological functions of mitochondria [155,156,157]. The copy number of mitochondrial genome in a typical mammalian somatic cell is approximately 2–5 × 10^3^, whereas the number of mtDNA molecules in a meiotically matured (MII-stage) oocyte is about 1.6 × 10^5^ in mice, 2.5 × 10^5^ in cattle, 3–5 × 10^5^ in pigs, and 3–8 × 10^5^ in humans. The number of mitochondria in the somatic cell averages 1 × 10^3^, and one organelle harbors between 1 and 10 mtDNA molecules. In turn, a single mitochondrion in the meiotically matured oocyte contains from one to two copies of the mitochondrial genome, which confirms that the abundance of the intraooplasmic population of these organelles is generally equivalent to the total pool of mtDNA molecules of an unfertilized mammalian oocyte [106,139,158,159].

In the procedure of cloning by SCNT, mitochondria of nuclear donor cells are transplanted with the nuclear genetic apparatus into the cytoplasm of enucleated recipient oocytes. Irrespective of the method used for the reconstruction of enucleated oocytes (Table 1), this step of the SCNT procedure always results in the conjunction and mingling (hybridization) of cytoplasmic environments of the ooplast and somatic cell or karyoplast. After its intraooplasmic microinjection, the karyoplast may also be a source of mitochondria (mitochondrial genome) of heteroplasmic origin. Therefore, a reconstructed cloned embryo, which from a cytological viewpoint is a cytoplasmic hybrid (cybrid), harbors the mitochondrial genome of both maternal (oocyte’s) and exogenous origin (i.e., introduced together with the nuclear donor cell) [107,111,160,161,162]. In cloned embryos, fetuses, and offspring, mitochondria are primarily inherited with ooplasmic material. In turn, probably during the first few mitotic cleavage divisions, mitochondria derived from nuclear donor cells are rapidly eliminated from the cytoplasm of embryonic cells at the anaphase stage. The removal of somatic cell-inherited mitochondria largely depends on the polyubiquitination of specific protein substrates. For that reason, the presence of the somatogenic mitochondrial genome in the cells of cloned blastocysts is difficult to detect by genetic engineering techniques [133,158,163,164]. As a consequence, the uniparental inheritance of extranuclear genetic information in dividing cybrid cloned zygotes is regulated by the biodegradation of ubiquitin-labeled mitochondrial proteins (including ribonucleoproteins) and the nucleolysis of mtDNA molecules that are deprived of histones and non-histone proteins. The proteolytic degradation of mitochondria of heteroplasmic (allogeneic) origin is catalyzed by a complex proteasomal system in each blastomere of cloned embryos. This system is characterized by a Svedberg sedimentation coefficient of 26 and designated as a 26S proteasome. The mechanism of nucleolytic biodestruction of all the somatic-cell-derived mtDNA copies is determined by normal function of the intracellular lysosomal cycle, which is related to the exocytosis of endosomal vesicles. The ultimate outcome of this reaction is the removal from embryonic cells of the exogenous mtDNA fractions, which had previously been subjected to internucleosomal fragmentation into short oligonucleotide segments. The preimplantation-stage selective segregation of the mitochondrial genome stemming from nuclear donor cells that is indirectly induced by the anaphase-promoting complex/cyclosome (APC/C) gradually leads to the establishment of cellular mtDNA homoplasmy in cloned embryos reconstituted with somatic cell nuclei. It is noteworthy that APC/C undergoes the heterodimerization with cyclin-dependent kinase cdc20 and is an integral part of the polysubunit enzymatic complex of ubiquitin ligase. Only occasionally could the lasting hybridization of allogeneic mtDNA copies (the so-called mtDNA heteroplasmy) be identified in the pre- and postnatal period of ontogenetic development of mammalian cloned specimens. This phenomenon of intracellular mtDNA heteroplasmy resulted from synergism/complementarity in the intergenomic communication between mtDNA molecules inherited with both nuclear donor cell cytoplasm and nuclear recipient cell ooplasm [155,165,166,167,168].

There are several species-specific epigenetic factors present in the oocyte cytoplasm that may contribute to nuclear–cytoplasmic incompatibilities either immediately after somatic cell nuclear transfer or at later stages of cloned embryo development [105,136,169,170]. In turn, this potential lack of coordination in the interactions of nuclear and cytosolic factors of cybrid cloned zygotes is probably one of the reasons for the limited practical application of the somatic cell cloning technique. It has been demonstrated that maternally inherited mtDNA molecules accumulated in the mitochondrial reservoirs of the oocyte cytosol play an important role in nuclear–ooplasmic asynchrony. This asynchrony involves incompatibilities in both the epigenetic modifications of the somatic genome supporting the developmental program of reconstituted cybrids and a lack of synergy in the molecular mechanisms controlling the karyokinesis and cytokinesis restriction points. These restriction points related to the anaphase segregation of somatic cell-derived chromosomes and asymmetrical telophase division of the cloned cybrid (nuclear–ooplasmic hybrid) that encompasses the expulsion of the pseudo-polar body into perivitelline space are collectively responsible for coordinated pseudomeiotic to mitotic cycle transition following activation of the reconstituted oocyte [134,156].

Moreover, the presence of an oocyte-derived mitochondrial genetic apparatus has been shown to influence the implantation of cloned embryos in the endometrium of a recipient female’s uteri. For that reason, the deleterious effect, on the preimplantation development of cloned embryos, of heterogeneous mtDNA sources as a result of possible mitochondrial heteroplasmy in the reconstructed nuclear–cytoplasmic hybrids should not be discounted [139,160,161,169]. That is why the production of nuclear-transferred embryos, fetuses, and offspring with a precisely defined profile of nucleotide sequences in regulatory or coding segments of the nuclear and/or mitochondrial genome seems to be valuable tool. This tool can be suitable for experimentally dissecting the effects of not only nuclear and cytoplasmic genetic/epigenetic components but also the intrauterine environment of recipient females on embryonic, fetal, and postnatal development of cloned specimens [2,155,158,171].

Therefore, in the hybrid cytoplasmic environment of cloned zygotes, genetically different fractions of mitochondrial DNA of maternal (oocyte’s) origin were found to coexist with those derived from the cytoplasm of allogeneic somatic cells. Although this extranuclear (mitochondrial) genetic apparatus of cloned nuclear–ooplasmic hybrids contains small (approximately 0.01%) amounts of a cell’s genetic information, this mtDNA-dependent genetic information is completely different from information recorded in the nucleotide sequences of nuclear DNA. The latter provides approximately 99.99% of the cellular genome. In this respect, nuclear transplantation of allogeneic somatic cells into enucleated recipient oocytes (where nuclear donor cells and oocytes are derived from genetically different animals of the same species) gives rise to generating nuclear–cytoplasmic hybrids, which are characterized by heterogeneous mtDNA copies. In view of the fact that such heteroplasmic cloned cybrids develop into embryos with cellular mtDNA heteroplasmy, this may lead to apparent genotypic and phenotypic identity/compatibility of the cloned offspring (only in terms of traits determined by nuclear genome-dependent inheritance). Such cloned offspring exhibits a degree of variation/incompatibility with regard to phenotypic traits determined by cytoplasmic (extranuclear) inheritance. The latter is dependent on the mitochondrial genotype known as the mitotype [105,157,159,160,167].

**Figure 1:**
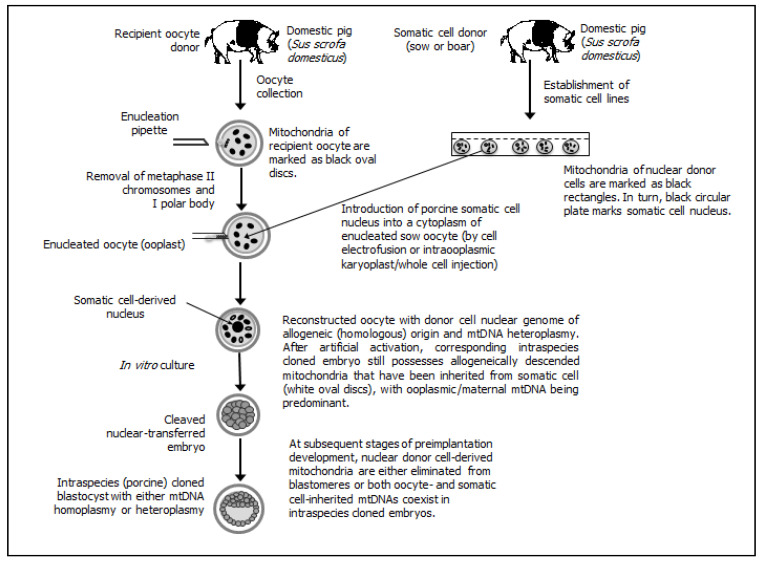
Intraspecies somatic cell cloning, in which the inheritance of allogeneic (homologous) mtDNAs stemming from genetically different nuclear recipient oocytes and nuclear donor cells is still incompletely recognized during preimplantation development of nuclear-transferred pig embryos. In the vast majority of intraspecies (porcine) cloned embryos, mitochondrial genome primarily arises from the nuclear recipient oocytes, whereas in their other counterparts, mtDNA copies appear to be inherited heteroplasmically (i.e., both from nuclear donor cells and from recipient ooplasm).

**Figure 2:**
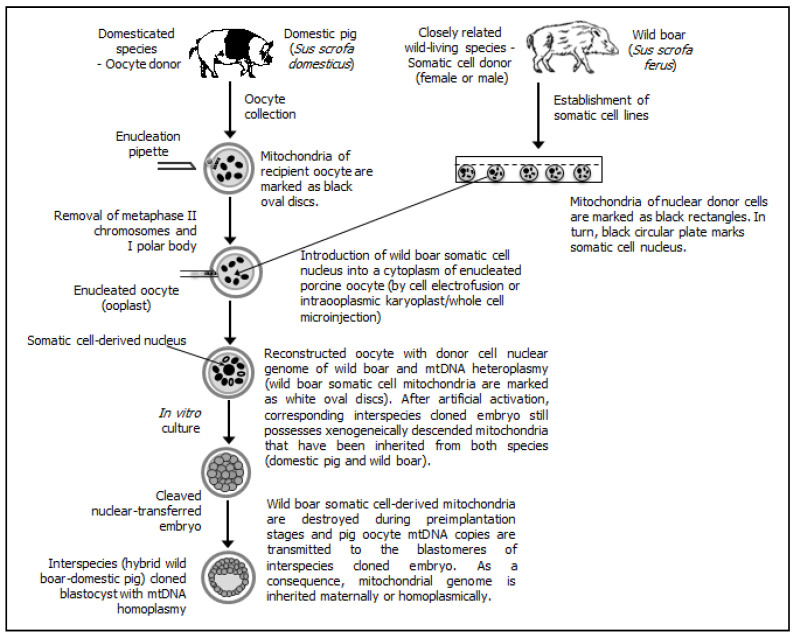
Interspecies (intrafamily and intragenus) somatic cell cloning, in which nuclear donor cells and recipient oocytes are recovered from phylogenetically consanguineous species (i.e., wild boar and domestic pig, respectively). Porcine oocyte-derived mitochondrial DNA (mtDNA) is inherited predominantly during preimplantation development of interspecies (wild boar→pig) cloned embryos, leading to intracellular mtDNA homoplasmy at the blastocyst stage.

**Figure 3:**
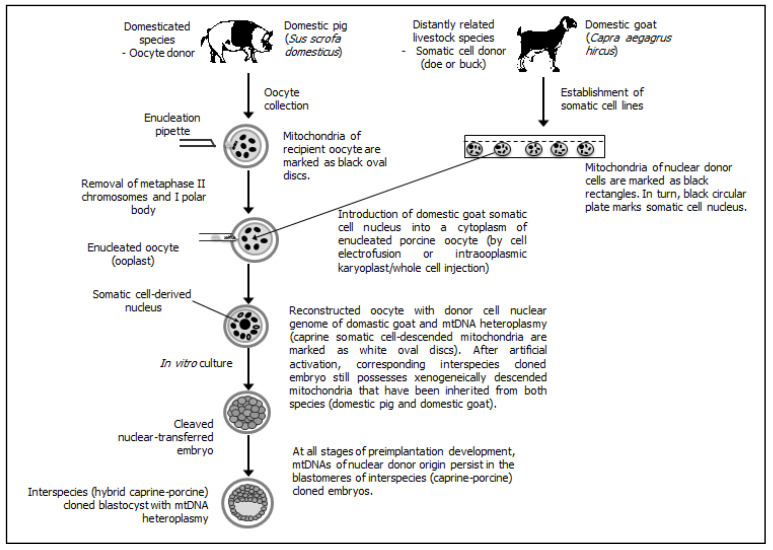
Interspecies (interfamily and intergenus) somatic cell cloning, in which nuclear donor cells and recipient oocytes are recovered from phylogenetically non-consanguineous species (i.e., domestic goat and domestic pig, respectively). Xenogeneic (heterologous) mitochondria that have been inherited from caprine nuclear donor cells and porcine recipient oocytes coexist during preimplantation development of interspecies (goat→pig) cloned embryos, leading to intracellular mtDNA heteroplasmy at the blastocyst stage.

Different possible patterns/scenarios of extranuclear (cytoplasmic) inheritance of mtDNA fractions have been presented (see Figure 1 (for intraspecies cloning by SCNT) [2,158,165,172], Figure 2 (for interspecies SCNT using nuclear donor cells and recipient oocytes derived from closely related mammalian species) [163,170,171,173,174], and Figure 3 (for interspecies SCNT using nuclear donor cells and recipient oocytes derived from phylogenetically distant mammalian species) [162,164,175,176]).

The “ideal” clone can be generated only in a situation where the nuclei of its own (autogeneic) somatic cells are transferred into enucleated recipient oocytes. Put another way, such a cloned specimen can be produced when nuclear donor cells and oocytes originate from genetically identical individuals of a mammalian species, i.e., from monosexual (female) individuals. It is necessary to stress that completely homoplasmic cybrid cloned zygotes can only be created from the oocytes reconstructed in such a manner. The latter are characterized by homogeneous fractions of mtDNA molecules. The artificial activation of such nuclear–cytoplasmic hybrids results in the development of cloned embryos displaying cellular mtDNA homoplasmy. This naturally results in complete genotypic and phenotypic identity/compatibility of somatic cell-cloned fetuses and the resultant offspring. Taking into consideration the previously mentioned findings, only in the case of mammalian cloned females does the mitotype exhibit a homogeneous pattern of coding and regulatory sequences in all mtDNA copies of the somatic and germ cell lines. This condition can only be met assuming that during ontogenesis, the mitochondrial genome will not undergo spontaneous point mutations or those induced by reactive oxygen species [2,42,106,133,166,168,172,177].

Among the reasons for genetic diversification between the cloned specimens generated (somatic clones) and individuals subjected to somatic cell cloning (i.e., donors of somatic cells for SCNT procedure), mention should be made of the effect of mitochondrial (extranuclear/extrachromosomal) inheritance and the impact of intrauterine environment of recipient females receiving cloned embryos. Extranuclear inheritance of genetic material results from the microsurgical, random introduction of foreign mtDNA copies with the nuclear donor cell cytoplasm into the cytoplasmic environment of recipient oocyte. The mismatch of the mitochondrial genome molecules of maternal (oocyte’s) origin and of somatogenic (nuclear donor cell) origin, i.e., mtDNA heteroplasmy, leads to inter-specimen diversification within the mitotype. This results in intra-population and inter-population genetic and phenotypic variability dependent on the mitochondrial genome [134,155,156,157,161]. The phenotypic differences between somatic clones and specimens undergoing SCNT are also contributed by different morphological, anatomotopographical, histological, physiological, endocrinological, embryotrophic, and immunological considerations associated with the reproductive system of recipient surrogates. Moreover, transplacental leakage of leukocyte and erythroblast mitochondria from the blood stream of recipient surrogates to the blood stream of cloned fetuses is often observed. This type of leukocyte–erythroblast chimerism results both from mtDNA heteroplasmy in peripheral blood cells and from genetic mosaicism within subpopulations of nucleated hematopoietic cells (i.e., hematopoietic karyocytes). Such chimerism may also have a certain effect on differences in the mitotype of cloned progeny [139,160,165,169].

## 4. Epigenetic Reprogramming of Telomeres in Chromosomes Inherited from Somatic Cell Nuclei throughout Development of Cloned Embryos, Fetuses, and Progeny

One of the essential prerequisites for epigenetic reprogramming of the cellular memory dependent on somatic cell-derived nuclear genome (nuclear DNA; nDNA) in the ontogenesis of mammals produced by SCNT is the structural–functional rearrangement of nuclear chromatin. The latter is associated with conformational changes in the length of terminal ends of chromosomes known as telomeres [178,179,180,181,182]. In turn, epigenomic biochemical alterations within telomeric chromatin are related to the biocatalytic activity of the telomerase enzyme [3,183,184,185]. One unresolved problem is the “epigenetic age” of cloned animals, which seems to be correlated to the length of terminal DNA fragments, i.e., the telomeres [186,187,188,189]. The telomeres are deoxyribonucleoprotein structures involved in the stabilization of the structure and conformation of nuclear chromatin during the division period of the mitotic cell cycle. This is necessary for the replication of mutation-free genomic DNA and karyokinetic segregation of chromosomes [190,191,192]. The replication of linear DNA in eukaryotic nuclear chromatin encounters the problem that the 5′-end of the lagging strand cannot replicate, as there is no space for the replication initiating RNA primer. An RNA primer is synthesized on the lagging strand template by primase or RNA polymerase, whose role is played by DNA polymerase α. This creates the risk that somatic cell chromosomes will shorten with every replication round, thus losing genetic information. In mammalian somatic cells, the classical α isoform of DNA polymerase is not capable of semiconservative replication of the 5′-end synthesized in fragments of the DNA chain, whose replication is delayed in relation to the 3′-end of the continuously copied leading strand [182,193]. As a result, in each cell division cycle, unreplicated telomere DNA sequences are gradually lost. For this reason, telomere length is a specific “physiological mitotic clock” of the cell. The shortening of chromosome telomeric regions is positively correlated with the number of cell divisions. Therefore, when the telomere length reaches a critical restriction/control point in a karyokinetically active somatic cell, this is signalized by the loss of nuclear chromatin stability, which is epigenetically programmed in the spatial structure/configuration and telomere functions. This is also signalized by triggering replicative senescence in the cell [187,194,195,196]. The characteristics of cells that undergo progressive replicative senescence include a considerable increase in diameter and a flattened shape caused by a drastic increase in cytosol volume. All of the above-mentioned epigenetic, genetic, physiological, morphological, and ultrastructural transformations, which occur in aging cells, lead in the first place to a rapid slowdown of both intracellular anabolic processes and the kinetics of mitotic divisions. At a later stage, these transformations bring about the irreversible inhibition of metabolic and proliferative activity. As a consequence of single doubling in the population of mammalian adult dermal fibroblasts cultured in vitro, telomere length decreases by about 48 DNA nucleotide pairs [180,183,188,197,198].

Telomerase is a ribonucleoprotein enzyme complex that displays the total activities of RNA reverse transcriptase and DNA integrase only in germ and embryonic cells, while its partial activity is observed in fetal somatic cells undergoing tissue-specific cytodifferentiation. However, the biocatalytic activity of this enzyme completely ceases in terminally differentiated somatic cells of adult specimens [184,185,193,199]. The function of telomerase is to restore the primary length of DNA telomeres by reverse transcription of its own RNA template. This gives rise to the de novo synthesis (reduplication) of tandem repeats within noncoding telomere DNA sequences (5′-TTAGGG-3′) that were lost as a result of terminating either consecutive mitotic and meiotic divisions of gametogenic (germinal) cells or mitotic cycles of blastomeres, leading to consecutive cleavage divisions of embryos. In the last phase of semiconservative DNA replication, the 3′-end of the leading strand extends beyond the 5′-end of the lagging strand. Telomerase contains an RNA molecule that is partially complementary to the tandem repeat of the short 5′-TTAGGG-3′ sequence at the 3′-end of the leading DNA strand, thus elongating the leading strand of telomeric DNA region using RNA as the template. Next, the enzyme detaches and binds to a new telomeric end to extend the leading DNA strand. The extension process may occur hundreds of times before telomerase finally dissociates. Then, the extended, replicated leading strand serves as a template for replication of the 5′-end of the lagging strand that is catalyzed by DNA polymerase α. These two processes, where the 5′-ends of DNA are shortened during basic semiconservative replication and subsequently elongated due to telomerase activity, are mutually balanced, whereby the total chromosomal length remains more or less the same [180,192,199,200]. In contrast, a lack of telomerase activity and, as a consequence, a lack of elongating the temporally and spatially restricted length of nuclear DNA telomeric sequences are epigenomically determined factors specific for adult somatic cells that provide a source of nuclear donors for the SCNT procedure. These factors limit the survival rate, proliferative activity, and the number of division cycles of a cell before the cell reaches the critical point of the maximum telomere shortening. The latter is simultaneously the mitotic control point that signals the initiation and irreversibility of the replicative senescence of terminally differentiated somatic cells [188,190,195,201].

The problem of telomere shortening/attrition and replicative senescence of somatic cells was observed in chromosomes of Dolly the sheep, the first cloned mammal [114,178,189,196]. The telomeres in the chromosomes of Dolly the cloned ewe (at the age of 3 years) were much shorter than the telomeres in the chromosomes of control animals, which were of the same age and were born through natural reproduction. Moreover, the telomere length in Dolly’s chromosomes was similar to that in the chromosomes of a 6-year-old sheep, which was used as a donor of somatic cells for the cloning procedure. At the time of molecular analysis of telomeres, Dolly was 3 years old, and her epigenetic age corresponded to the actual age of a 9-year-old sheep. Put differently, Dolly’s somatic cells were epigenetically older by 6 years than herself. Born on 5 July 1996, Dolly the sheep lived above 6.5 years and was euthanized on 14 February 2003 after being diagnosed with a malignant lung cancer known as Jaagsiekte (ovine pulmonary adenocarcinoma). The etiologic agent of this chronic, contagious, and fatal lung cancer in sheep is Jaagsiekte sheep retrovirus (JSRV), which is responsible for the oncogenic transformation of bronchial exocrine epithelial cells, i.e., type II pneumocytes and bronchiolar club (Clara) cells. By 2000, Dolly produced a total of 6 lambs (including twins and triplets). Therefore, the cloned ewe was reproductively sound and displayed high fertility and prolificacy, which means that her reproductive capacity upon reaching sexual and breeding maturity was not impaired. However, in 2001, the hind legs of 5-year-old Dolly exhibited the first symptoms of an autoimmune chronic degenerative joint disease (osteoarthritis), namely rheumatoid arthritis. It should be noted that this disease is relatively frequent in different breeds of sheep, but generally, it does not affect animals younger than 10 years of age. Two questions arise: Could Dolly live 6–9 years less than the expected lifespan of 12–15 years (which is the average lifespan of Finn Dorset sheep, represented by the somatic cell donor ewe in the SCNT procedure)? As a result of somatic cell cloning, did she exhibit rapidly progressing symptoms of premature (anatomical and physiological) aging of the entire body or of some of its parts, tissues, and organs? The results of experiments performed to determine the telomeric age of Dolly the sheep suggest that animals cloned by transferring adult somatic cell nucleus into the enucleated oocyte are epigenetically compromised. For this reason, they have a genetic age of a specimen playing the role of somatic cell donor for SCNT. This means that at birth, they are epigenetically and genetically much older than their real-time birth date [178,184,189,196]. However, the evidence for cloned sheep was not reflected in the studies focused on the analyses of chromosomes isolated from somatic cells derived from cloned cattle. A study by Lanza et al. [194] on the chromosomes of cloned calves produced by using long-term cultured fibroblast cells for SCNT showed that the telomere length of these young animals is even slightly greater than that of control animals, despite the fact that the chromosomes of nuclear donor cells were almost completely depleted of telomeres. These analyses confirmed that the terminal ends of chromosomes are efficiently resynthesized in blastomeres of bovine cloned embryos, with a contribution from highly active telomerases. Analogously, Tian et al. [179] demonstrated that telomere length in chromosomes of four live (about 15.4 kbp) and six dead cloned calves (about 15.9 kbp) that had been generated using dermal fibroblast cells or cumulus cells derived from a 13-year-old cow not only did not differ considerably from the telomere length characteristic of control chromosomes (about 14.7 kbp) but also significantly exceeded (by about 3–3.5 kbp) the telomere length in chromosomes of the aging cow (about 12.4 kbp), which served as the donor of somatic cells for SCNT-based cloning. Finally, Kato et al. [202] provided evidence that in cloned cattle, the telomere length is shortened only in the tissues matching those from the biopsy specimens of which the primary cell cultures were established. In turn, the latter provided the somatic cell lines that were nuclear donors used for SCNT procedures.

Telomere length in chromosomes of the dermal fibroblast cells originating from six transgenic cloned pigs matched the telomere length of the chromosomes of dermal fibroblast cells originating from control animals of the same age and produced by natural reproduction. In turn, two cloned piglets that died 3 to 7 days after birth displayed the same length of terminal ends of chromosomes as the telomeres of chromosomes in fetuses at the third trimester of pregnancy [185]. The terminal restriction fragment (TRF) assay of genomic DNA isolated from the cells stemming from the biopsy specimens retrieved from different organs/tissues of cloned fetuses (gonads, heart, liver, lungs, kidneys, and skin) has confirmed that telomere length in chromosomes remains constant in all the cell lines arising from cytodifferentiation that takes place throughout fetogenesis. The reason for this is the high efficiency of restoring the primary length of terminal chromosome ends via the active telomerase isoform throughout the interphase replication cycle of nuclear DNA in differentiating somatic cells that occupy new tissue niches and are engaged in multi-stage histo- and organogenesis processes. During the postnatal period, telomeres are gradually shortened with each mitotic division of somatic cells, and the reduction of telomere length is tissue-specific. This reflects inhibition of the biocatalytic activity of telomerase in differentiated lines of somatic cells derived from skin tissue explants and various internal organs harvested from gilts and boars both before and after attainment of sexual maturity [180,181,185,192,198].

## 5. Comprehensive Summary and Future Goals

Cloning by SCNT is currently used in assisted reproductive technologies (ARTs) of many mammalian species, including various species of farm animals. The application of this technology in experimental embryology and in molecular population genetics is of great importance for livestock breeding.

Somatic cell cloning as a method of asexual reproduction offers the opportunity for production and/or multiplication of monogenetic and monosexual progeny of high breeding worth, whose genotypic and phenotypic identity with progenitor donor of transcriptional mitochondrial and nuclear apparatus of the somatic cell only concerns genomic DNA. Animals produced by SCNT differ in phenotypic traits determined by the random segregation of oocyte-derived/maternal and somatic cell-derived/somatogenic mitochondrial genome (mtDNA) as a result of cytoplasmic (extranuclear) inheritance of genetic material [106,133,159,161]. Nevertheless, the particularly high application value of somatic cell cloning technology is related to the possibility of generating genotypically and phenotypically identical transgenic animals, i.e., animals with transformed nuclear genomes that are valuable due to the expression product of modified genes [4,28,38,203]. The yield of recombinant transgenic protein synthesis by genetically transformed cloned specimens is, to a certain extent, dependent on the effect of heteroplasmic sources of mitochondrial genotype (mitotype) on the transcriptional activity profile of modified nuclear DNA genes. This correlation may be negative with a high coefficient of heritability and regressive repeatability of a given quantitative and qualitative trait resulting from the transgenization of a breeding herd [2,133,158]. Therefore, an important problem in the production and multiplication of transgenic cloned specimens (the so-called clonal founder animals) is to generate offspring with an identical mitochondrial genome. These offspring carry only homoplasmic copies of mtDNA derived either from recipient oocytes or from somatic donor cells of genetically modified nuclei. Not without significance is the effect of inheritance of extranuclear genetic information that is accumulated in mitochondrial reservoirs of both somatic (somatogenic) and germinal (gametogenic) cell lines on the transcriptional activity of quantitative trait loci (QTLs). The latter encompass loci for such traits of transgenic cloned specimens as reproductive traits (e.g., fertility and prolificacy), productive traits, including meatiness (e.g., loin eye area, contents of striated muscle tissue, intramuscular and intermuscular connective tissue, adipose tissue in different carcass, and half-carcass cuts) and milk yield traits (e.g., volume of milk synthesis, and milk secretion and ejection per day and per lactation period) [2,4,106,108,136,157,159,203]. In turn, genetic determinants of prolificacy or fertility traits from the heteroplasmic or homoplasmic pattern of mitochondrial genome segregation may influence the processes of intergenerational transmission of the transgene in germ cell lines of the descendant generations of cloned animals with the transformed nuclear genotype. On the one hand, a negative or positive genetic correlation between milk yield or dressing percentage traits (inherited with genomic DNA) and the transcriptional activity profile of mitochondrial DNA genes may be responsible for different extents or patterns of tissue-specific or organ-specific expression of xenogeneic (e.g., human) gene constructs in transgenic cloned animals. The expression extents or patterns of these gene constructs may be characterized by the inhibition or onset of their transcriptional suppression. On the other hand, the above-mentioned negative or positive correlation may also affect the expression profile of xenogeneic gene constructs (transgenes) in different cells, tissues, and organs of genetically modified cloned specimens. This profile of transcriptional activity of the transgenes integrated with the nuclear genome may be homogenous or heterogeneous, resulting in the induction or absence of transgenic mosaicism/chimerism in cloned animals [4,5,136,158,168,169]. The xenogeneic expressive gene constructs that have been incorporated into genomic DNA of cells localized in different tissues and organs of transgenic cloned animals can encode, for example, recombinant human therapeutic proteins. The synthesis and exo- or endocrine secretion of these proteins can be targeted at secretory cells of the mammary gland or smooth and striated muscle tissue found in all the corporeal organs, organ systems, and parts of farm animals [35,139,204,205,206,207].

Intergenomic communication between mitochondrial DNA and the transgene stably integrated with nuclear DNA may also create differences in the efficiency of transgenesis, which induces targeted mutagenesis, i.e., monoallelic deletion or the insertional inactivation of the gene coding for myostatin. Myostatin is a muscle-tissue-specific hormonal protein that paracrinally inhibits the gain (hypetrophy and hyperplasia) of skeletal and smooth muscles [18,208]. The presence of one or two knockout alleles of the myostatin gene or the presence of one or two posttranscriptionally silenced mRNA copies encoded by the myostatin gene in heterozygous or homozygous transgenic cloned beef cattle increases meatiness in cows and bulls. This results from the hypertrophy and hyperplasia of not only striated but also smooth muscle tissue [18,204].

The attractiveness of SCNT-based cloning of transgenic mammals, including various species of domesticated animals, is decided by the applicability of the hormonal or enzymatic product of the modified gene expression. This applicability first of all determines the scale and scope of the research. Although the first cloned mammal was a sheep, research targeted at the somatic cell cloning of other livestock species had a much wider span. The mammary glands (udders) of transgenic cloned cows [11,12,14,206,209,210], transgenic cloned sheep [23,24], and transgenic cloned goats [17,19] may become live bioreactors for producing humanized milk, easy-to-digest milk, or milk containing recombinant human therapeutic proteins (biopharmaceuticals or nutraceuticals). The latter may find clinical application in the treatment of patients afflicted with genetically determined diseases [12,211,212,213].

Compared to other ARTs in mammals (including livestock species), the efficiency of somatic cell cloning in domesticated animals, which is measured by the percentage of offspring born in relation to the number of reconstructed oocytes, remains low and oscillates between 0.3% and 2% on average. Nonetheless, the biotechnological possibilities of the somatic cell cloning in different mammalian species is far ahead of our understanding of the biological determinants, in particular the molecular and epigenetic aspects, of this method [108,132,192,203]. Yet, the biological foundations that have been laid for embryonic genome engineering of domesticated animals, especially over the last 24 years, made feasible the development of an innovative technology of in vitro embryo production using the somatic cell cloning procedure, which may meet the requirements for application in laboratories or, in some cases, only for limited practical purposes [127,214,215]. The assisted reproductive technology that encompasses SCNT could be used on a larger practical scale only after the efficiency of somatic cell cloning in various mammalian species, including livestock, is increased to match the efficiency of in vitro fertilization (IVF) or artificial insemination (AI) as part of multiple ovulation and embryo transfer (MOET) programs in cattle. However, due to the relatively high incidence of lethal or sublethal developmental anomalies or anatomo-histological defects in cloned fetuses and progeny, it is not possible to use the somatic cell cloning of farm animals on a commercial scale, at least at the present level of sophistication of the relevant research performed in Europe and the world. Furthermore, it is also worth noting that the elaboration and optimization of efficient approaches applied to cryopreserving nuclear donor somatic cells, nuclear-transferred oocytes reconstructed with somatic cells, and somatic cell-cloned embryos appear to be important milestones that can help cryogenically protect these valuable types of biological materials. This can bring the investigators closer to the perspective of progression in the outcome of producing mammalian SCNT progeny. In turn, future large-scale attempts undertaken to more successfully generate mammalian cloned offspring can expedite their practical use for the purposes of not only agricultural, transgenic, biotechnological, biomedical, and biopharmaceutical research fields but also ex situ conservation of biological diversity in different anthropogenic and unspoiled natural ecosystems.

To sum up, it seems that after making the transition from basic to applied research, the techniques for intra- and interspecies somatic cell cloning of mammals could contribute to (1) the conservation of genetic resources and the establishment of the genetic reserves of threatened mammalian species and breeds, (2) the restoration and multiplication of the subpopulations of endangered or vulnerable wild and domesticated species of mammals in order to maintain biodiversity and to increase the level of intra-population and inter-specimen genetic variability, and (3) revival (“resurrection”) and reintroduction into the wild of extinct, free-living species of mammals. Moreover, the practically applied research into the cloning of domesticated animals could serve to achieve other tangible benefits, including (4) the improvement of the breeding (genetic) and productive value of different farm animal breeds, e.g., increasing their milk and meat yields and reproductive ability (prolificacy and fertility), and (5) the implementation of basic research into interdisciplinary sciences aimed at the generation of animal biotechnological (transgenic) products for the biomedical, biopharmaceutical, nutraceutical, and food technology industries. One classic example of this is the permanent and highly heritable targeted transgenization of the mammary glands of domesticated species of small and large ruminants (i.e., sheep, goats, and cattle, respectively) and their use as animal bioreactors for producing humanized milk or milk containing recombinant human therapeutic proteins such as biopharmaceuticals and nutraceuticals.

## Figures and Tables

**Table 1:** Comparative characterization of the approaches to reconstruction of enucleated mammalian oocytes at the biotechnical, cytological, molecular, and epigenetic levels.

Method Used for Reconstruction of Enucleated Metaphase II-Stage Oocytes	Characterization at the Biotechnical and Cytological Levels	Characterization at the Molecular Level	Characterization at the Epigenetic Level
**Electrofusion of ooplast–somatic cell couplets**	Relatively low invasiveness of the method: - the generated electrostatic field interferes with ultrastructure and functions of the oolemma of nuclear recipient cells and the plasmalemma of nuclear donor cells through: • transient formation in the plasma membrane phospholipid bilayer of micropores (microchannels) that facilitate fusion of ooplast–somatic cell complexes and are the pathway for passive intracellular transport of calcium ions under the conditions of simultaneous fusion and electrical activation (F/A) of reconstituted oocytes	Relatively high probability of the occurrence in the obtained clonal cybrids of: - cellular mtDNA heteroplasmy; - abnormal nuclear–cytoplasmic interactions; - abnormal intergenomic communication between allogeneic nuclear DNA, somatic cell-inherited mtDNA molecules, and mtDNA molecules of ooplasmic origin	Relatively high probability of the occurrence of abnormalities in: - structural and epigenetic remodeling of nuclear chromatin; - epigenetic reprogramming of transcriptional activity of the nuclear genome, including rearrangement of telomeres of somatic cell chromosomes in the reconstructed oocytes and in cloned embryos that develop as a result of their activation
**Direct intraooplasmic** **microinjection of whole somatic cells**	High invasiveness of the method: - interferes with the ultrastructure of the plasmalemma and the membrane and cytoskeleton of enucleated oocytes through: • direct microsurgical transfer and deposition in their ooplasm of tiny (small-diameter) somatic cells displaying intact integrity of the plasma membrane	Relatively high probability of the occurrence in the obtained clonal cybrids of: - cellular mtDNA heteroplasmy; - abnormal nuclear–cytoplasmic interactions; - abnormal intergenomic communication between allogeneic nuclear DNA, somatic cell-inherited mtDNA molecules, and mtDNA molecules of ooplasmic origin	Relatively high probability of the occurrence of abnormalities in: - structural and epigenetic remodeling of nuclear chromatin; - epigenetic reprogramming of transcriptional activity of the nuclear genome, including rearrangement of telomeres of somatic cell chromosomes in the reconstructed oocytes and in cloned embryos that develop as a result of their activation
**Direct intraooplasmic microinjection of karyoplasts**	The highest invasiveness of the method: - interferes with the ultrastructure of the plasmalemma and the membrane and cytoskeleton of nuclear donor cells through: • their mechanically induced cytolysis to isolate karyoplasts - interferes with the ultrastructure of the plasmalemma and the membrane and cytoskeleton of enucleated oocytes through: • direct microsurgical transfer and deposition in their ooplasm of karyoplasts	Relatively low probability of the occurrence in the obtained clonal cybrids of: - cellular mtDNA heteroplasmy; - abnormal nuclear–cytoplasmic interactions; - abnormal intergenomic communication between allogeneic nuclear DNA, somatic cell-inherited mtDNA molecules, and mtDNA molecules of ooplasmic origin	Relatively low probability of the occurrence of abnormalities in: - structural and epigenetic remodeling of nuclear chromatin; - epigenetic reprogramming of transcriptional activity of the nuclear genome, including rearrangement of telomeres of somatic cell chromosomes in the reconstructed oocytes and in cloned embryos that develop as a result of their activation

## Abbreviations

AI	Artificial insemination
APC/C	Anaphase-promoting complex/cyclosome
ARTs	Assisted reproductive technologies
IVF	In vitro fertilization
JSRV	Jaagsiekte sheep retrovirus
MII	Metaphase II
MOET	Multiple ovulation and embryo transfer
mtDNA	Mitochondrial DNA
nDNA	Nuclear DNA
QTLs	Quantitative trait loci
SCNT	Somatic cell nuclear transfer
